# The image of autism in the Polish press 2009–2020 and the role of first-person testimonies

**DOI:** 10.1186/s12888-024-06374-y

**Published:** 2025-02-17

**Authors:** Maciej Wodziński, Natalia Kamińska, Marcin Moskalewicz

**Affiliations:** 1 Philosophy of Mental Health Unit, Department of Social Sciences and the Humanities, Poznan University of Medical Sciences, Rokietnicka 7, Poznan, 60-806 Poland; 2 Institute of Philosophy, Maria Curie-Skłodowska University, M. Curie-Skłodowska sq. 5, Lublin, 20-031 Poland; 3 International Cooperation Center, Maria Curie-Skłodowska University, M. Curie- Skłodowska sq. 4, Lublin, 20-031 Poland; 4 Phenomenological Psychopathology and Psychotherapy, Psychiatric Clinic, University of Heidelberg, Heidelberg, Germany; 5 IDEAS NCBR, Chmielna 69st, Warsaw, Poland

**Keywords:** Autism, Neurodiversity, Discourse, Media representation, Epistemic injustice, Experts by experience

## Abstract

This study examines the representation of autism in Polish press discourse from 2009 to 2020 in five major Polish daily newspapers, specifically focusing on the influence of first-person testimonies of autistic individuals. Quantitative discourse analysis was conducted using deductive coding of 1352 press articles concerning ASC. In analyzed press reports, autism was primarily portrayed as a negative and deficit-laden phenomenon that should be addressed by bringing autistic people in line with the neurotypical majority, reinforcing social stereotypes and stigma. Autistic people’s perspective was mostly neglected. However, when autistic people’s voice and first-person perspective was presented, as in the case of 3.7% of the analyzed media, such stereotypical and negative perspectives on ASC significantly changed for the better.

Research outcomes highlight the disparities in autism portrayal, particularly contrasting the deficit-driven narratives with perspectives emerging from the inclusion of first-person narratives. The latter can mitigate harmful stereotypes and promote a more accurate and positive understanding of autism. Treating autistic people as an essential source of information about themselves significantly changes the press image of autism and may indirectly contribute to their quality of life.

## Background

The stereotypical ways of representing the Autism Spectrum Condition (ASC) influence the perception of autistic people by society and the construction of their individual and social identities. The public discourse has prioritized non-autistic people’s perspectives on autism. Autism has been primarily presented as a deficit-based condition aligned with its psychiatric medical model as a mental disorder. The dominant discourse has often side-lined other perspectives, such as that of the social disability model and the neurodiversity movement approach, which present autism as an alternative mode of lived experience. Consequently, the public image of ASC today is often harmful or biased.

Many discourses pervasive in public consciousness establish stereotypical views of phenomena and thus influence how one perceives the world [1, 2]. Main narratives (including media discourse) construct the public imagination of phenomena like autism and can lead to the reinforcement of stereotypes, prejudices, and other non-rational beliefs [3] or to unjust practices against mental health system users [4]:

‘(…) hermeneutical practices play an important role in health care because they allow service users sense-making reflectivity, which helps to turn a confusing and troubling set of symptoms into a more comprehensible and tenable context‘ [5] (p. 169).

Although any discourses, especially popular ones, portray the world from a particular perspective, these perspectives must not necessarily be harmful or biased. They may consider the actual state of affairs, avoid perpetuating negative stereotypes and prejudices, and express a plurality of possible opinions on the subject. However, this is not the case of the public image of Autism Spectrum Condition (ASC)^1^ [6, 7](see Table 1).

The analyzed media reports contribute to the formation of stereotypes because they are widely read by a general population of non-specialists, for whom these reports are often the only source of information on neurodiversity. If they portray autism through a deficit-driven, medicalized lens, focusing on challenges, abnormalities, and extreme cases, it reinforces negative stereotypes and stigma. The exclusion of autistic voices in favor of neurotypical perspectives and sensationalized stories further marginalizes autistic individuals, contributing to public misunderstanding.

A) Dominant discourses marginalize the voice of autistic people^2^ [8]. Most media statements say something “about them” but do not consider their first-person perspective [9, 10]. This research showed that allowing autistic people to speak in the public discourse can significantly improve its quality in terms of scope and diversity by bringing an invaluable first-person perspective on autism that is not otherwise available.
B) Dominant discourses impose the deficit perspective, that is, portray autism primarily through the lens of the problems, misfortunes, tragedies, and difficulties faced by autistic people and the rest of society [11]. In addition, autism research discourse can be biased in terms of ableism, that is, dehumanizing and treating autistic people as research objects [12].
C) Dominant discourses use medical and not social narration. They build a fundamental narrative around health problems and research to solve them [13]. They present autism as a medical problem, not an alternative experience mode [14]. These stereotypical ways of autism representation influence the perception of autistic people by society and the construction of their individual and social identities [15]. Furthermore, they affect the work of health professionals [16, 17]. Importantly, distinguishing between the voices of experts (health professionals and others) and those of individuals on the spectrum is crucial, as each offers different insights. Without clarifying this distinction, media reports risk perpetuating a one-sided, deficit-driven narrative that reinforces societal stigma. The opinions of experts can contribute a lot of relevant content, as people on the spectrum themselves do not always have up-to-date scientific knowledge to pass on to the public through the media (unfortunately, neither do experts always). However, autism is a phenomenon about living people, their lives, and their social experiences, and therefore it should not be portrayed mainly as a ‘research subject’ The narratives that make up the discourse should also include an appropriate proportion of ‘lived experience’ to help understand the way people on the spectrum experience the world. People on the spectrum are the only ones who can provide this kind of narrative. A balanced discourse on autism should include narratives from different groups in society.

Our study focuses on news media rather than social media, which is primarily due to their higher epistemic authority as well as the temporal scope of the research, which goes back 15 years when social media channels were not as developed as today.

**Table 1:** Main categories of popular discourses on ASC from previous studies (USA/GB/Australia/China)

Category	Research
Increase in the number of articles over the years	Wolbring & Mosig [11], Bie & Tang [18], Karaminis et al. [19]
„Missing voices” – autistic voices marginalized in the discourse	Bie & Tang [18], Clarke [20], Huws & Jones [9], Baroutsis et al. [21], Mittmann et al. [22]
Negative and deficit perspective on autism, stigmatisation cues	Wolbring & Mosig [11], Holton et al. [23], Huws & Jones [9], Jones & Harwood [14], Karaminis et al. [19], Lewin & Akhtar [24], Yu & Farrell [25], Mittmann et al. [22]
Medical narration shows ASC as mainly a health problem, marginalizing social frame	Wolbring & Mosig [11], Billawalla and Wolbring [13], Huws & Jones [9], Robertson [26], Karaminis et al. [19], Baroutsis et al. [21]
Autism presented a problem and not alternative way of experiencing the world	McKeever [27], Karaminis et al. [19]
Press representation biased toward males and children	Karaminis et al. [19]
Texts do not demonstrate high content focus on autism	Baroutsis et al. [21]

Media reports play a critical role in shaping public stereotypes through specific psychological mechanisms. For example, agenda-setting, a mechanism responsible for directing public attention to certain aspects of the phenomenon and overlooking others, by focusing attention on particular issues, such as autism-related challenges, while neglecting the perspectives of autistic individuals, thus shaping public priorities around the deficits, challenges, ‘fighting’ with autism, creating and reinforcing stereotypical views [28]. Framing theory drives this further, explaining, not only ‘what’ the audience thinks about, but also ‘how’ it thinks. The media emphasizes certain aspects of autism, such as deficits or medicalization perspectives, which influences how the public interprets the condition, often reinforcing negative stereotypes [29, 30]). Which ‘framework’ of portrayal of a given phenomenon is chosen by a given media outlet is the result of a variety of factors, ranging from current trends to the impact of the diffusion of scientific knowledge from various centres into the media and then further into society, to the effectiveness of the lobbying activities of autism-related communities. What is important, empirical evidence shows that stories focused on positive aspects of a condition can lead to more nuanced and positive portrayals of mental conditions, thus reducing stigma [31].

Press coverage plays a significant role in shaping societal perceptions of minorities, often reinforcing stigma and perpetuating harmful stereotypes. Research on mental illness demonstrates how biased or sensationalized reporting can influence public attitudes, further marginalizing already stigmatized groups. For instance, Whitley and Wang [32] revealed that Canadian newspapers have historically framed mental illness in negative or deficit-based terms, associating individuals with violence, unpredictability, or helplessness, which heightens public fear and misunderstanding. Similarly, Corrigan et al. [31] found that news stories emphasizing dangerousness or criminality increase stigmatization, while those focusing on recovery or success stories can reduce public bias.

For marginalized groups such as people with mental conditions, media depictions often simplify complex realities, relying on stereotypes that dehumanize or pathologize these individuals. According to Ramasubramanian and Yadlin-Segal [33], this leads to a cycle where public discourse about minorities is dominated by sensationalist headlines and deficit-focused narratives, reinforcing societal prejudices. Studies like Ischebeck et al. [34] on media portrayals of stigmatized minorities, such as people with pedophilic interests, illustrate how negative coverage can further alienate and dehumanize such groups. This trend not only deepens social divides but also limits opportunities for these communities to be understood in more nuanced or empathetic ways. Therefore, reshaping media narratives to include more diverse and positive representations is essential for breaking down stigmas and fostering societal acceptance.

### Polish context

Poland is where Eastern and Western European intellectual streams collide and mix [28]. While not being the vanguard of the neurodiversity movement, it assimilates and adapts the new trends to its needs. A non-medicalized view of autism has gained popularity and is increasingly present not only in the popular discourse but also in professional discussions. To date, however, there has been no scientific research on media representation of autism in Poland – a gap that this study aims to bridge.

Aside from a few initiatives at the governmental level (such as the Parliamentary Group for Autism, the Charter of Rights for People with Autism, or the Strategy for People with Disabilities 2020–2030), the vast majority of educational social actions are initiated by non-governmental organizations, such as Alpha Foundation, Prodeste Foundation, Autism Team Foundation, and Aware Youth Club, unlike in Australia, Canada, or the UK. Programs aimed at the vocational activation of autistic individuals indicate that employers are becoming more aware of the benefits of the complementary use of their potential. Self-advocacy activities of autistic people (like Jan Gawroński or Joanna Ławicka) have also visibly increased in the Polish public space, but opinion polls still confirm the presence of numerous stereotypes about autism [35]. Moreover, research shows that harmful stereotypes about autism persist despite the increase in awareness [29, 30]. The mental health care system has been in crisis for years, largely due to the drastically low number of specialists in the field of psychiatry, especially child psychiatry – a profession practiced by 564 individuals (as of September 2024), which translates to 1 per 12,000 children (parliamentary interpellation no 31560 [36]). One consequence is the lack of experts who could professionally shape the discourse [37–39].

### Methods

#### Sample

This research is based on the purposive sample of 1352 press articles concerning this topic and the first from Central Eastern European countries (for similar research from other regions see Table 2).

**Table 2:** Research on autism representation in press

Reseach	Location	Sample size (*n*)
Huws, Jones 2010 (9)	UK	255
Karaminis 2023 (19)	UK	23 742
Pesonen et al. 2020 (37)	Finland	210
Bie, Tang 2014 (18)	China	795
Wolbring, Mosig 2017 (11)	Canada	359
Jones, Harwood 2009 (14)	Australia	1228
Baroutsis et al. 2021 (21)	Australia	1351
Wendorf, Yang 2017 (30)	USA	413
McKeever 2013 (27)	USA	2185
Yu & Farrell 2020 (25)	USA	982
Lewin & Akhtar 2021 (24)	USA	315

We selected 2,180 press articles concerning autism published in the years 2009–2020 in five major all-national Polish press titles most influential by print circulation and the number of cross-media references: Gazeta Wyborcza, Rzeczpospolita, Gazeta Prawna, Fakt, Super Express (circulation statistics for individual dailies is available at the Polish Readership Survey at https://www.pbc.pl/). 2009 was chosen as it was the first year, in which articles on autism appeared in all five selected dailies. We searched each newspaper’s online archive for the phrase ‘autyzm’ (eng. ‘autism’), and ‘autystyczny’ (eng. ‘autistic’). The Polish word ‘autyzm’ (eng. ‘autism’) morphologically is a part of almost all variants of this word, for example, ‘zaburzenie ze spektrum autyzmu’ (autism spectrum disorder) or ‘osoba w spektrum autyzmu’ (a person on the autism spectrum), etc. For this reason, a search in the newspapers archives using the phrase ‘autyzm’ (without quotation marks) returned articles in which all variations of this word were used. In our search, we also used the phrase ‘autystyczny’ (autistic) and several of its morphological variants. However, all the articles found using this phrase that met the substantive criteria for inclusion in the analysis were duplicates of those retrieved by the phrase ‘autyzm.’ We downloaded all the relevant articles and excluded those that did not contain relevant content (such as advertisements, descriptions of TV programs, links to other press materials, articles on other topics, and duplicates). The final sample consisted of 1352 articles.

#### Research questions

The research was guided by the following questions:

1. What is the general tone of the discourse on the autism spectrum in the Polish press?
2. Is there a change in this respect over the analysed period?
3. What thematic frames are used when writing about the autism spectrum in news reports?
4. Which social groups speak out in articles on autism?
5. Of all the people speaking in the articles, what proportion are people on the autism spectrum and what proportion are health professionals?
6. How many of the articles analysed contain information that may help to understand the specific way in which people on the spectrum function and experience the world?
7. How many of the articles use deficit language, portraying autism as a difficulty and a problem?
8. How does the inclusion of first-person testimonies from autistic individuals influence the tone and framing of media articles about autism?
9. How do the perspectives of health professionals differ from those of autistic individuals in media coverage, and what impact does this have on the representation of autism?
10. Did the frequency of giving voice to people on the autism spectrum increase during the analyzed period?

#### Coding

We coded the articles’ main thematic frame (as either: medical, institutional, news and criminal cases, infrastructure, social functioning, economic, charity, celebrities, family situation, culture and art, misuse, other), their overall tone (as either: positive, negative, neutral, positive and negative), the voices included (as either: autistic people, health professionals, journalists, family), and the image of autistic people presented (as either: the text emphasizes the potential of autistic people, the need to change them to fit neurotypical society, or the need to raise social awareness and tolerance). Further on, we compared the contents of the articles depending on whether they included autistic people’s first-person perspective or all other groups, journalists, parents, community activists, scientists, etc. Separately, we compared autistic people with health professionals – a group defined as comprising medical doctors (including psychiatrists), psychologists, therapists, and social workers coming in contact with autistic people.

Some of the main coding categories were drawn from previous studies to allow comparison between countries. The list of thematic frames and the ‘who speaks’ group was pre-established based on previous and similar press discourse research in other countries (see: Billawalla and Wolbring 2014, Huws and Jones 2010, Bie and Tang 2014). The list was further expanded as previous studies did not consider many important and often recurring themes. This was perhaps due to differences in content or self-imposed limitations.

For example, we have added the ‘health professionals’ group to recognize their perspective, as this group holds a lot of ‘power’ over autistic people’s lives. We also added the category of neurodiversity lens, as the topic of neurodiversity was beginning to gain more attention at that time in Polish media. For details regarding codes and examples see Table 3.

**Table 3:** Codes with descriptions, percentages, and examples of quotes

Code	Description	% (*n*)	Example
		(*n* = 1 352)	
**Main thematic frame**	**The main theme around which the story is focused.**		
Medical	Autism shown as a medical phenomenon, e.g. therapies, research into causes of occurrence, potential ‘cures’, symptoms etc.	22.00% (298)	‘Sick children without a specialist. Parents of children with autism, ADHD or other disorders have nowhere in Radom to go for help because there is a shortage of child psychiatry specialists. - ‘We are completely helpless, relying only on ourselves.’’ ‘We are ruled by the microbiome. ' […] Other studies show that mice suffering from a type of autistic disorder similar to humans have a significantly lower abundance of the common gut bacterium Bacteroides fragilis than healthy mice. The animals were stressed and antisocial.’ ‘Monkey key to autism. The Chinese have created genetically modified macaques that will be used to study human developmental disorders. Until now, disorders of this kind have been studied using laboratory mice. Due to their different conformation and social behaviour, incompatible with humans, scientists could learn little from such experiments. The achievement of specialists from the Department of Neurology at the Shanghai Institute of Biological Sciences makes it possible to trace this mechanism in our cousins. The plan is to discover the causes of autism - and possible therapies.’
News and criminal cases	Stories about recent events involving autistic people, written in an emotionally compelling way to capture the reader’s attention or criminal cases involving autistic people.	15.8% (214)	‘Autistic 13-year-old shot repeatedly by police. He is fighting in hospital for life. Linden Cameron (13 years old), who has Asperger’s syndrome, was taken to hospital in a serious condition with bowel, bladder, shoulder, and ankle injuries after Friday’s shooting in Glendale. The perpetrators of the shooting were police officers who arrived to help the boy manage his mental breakdown.’ ‘A 10-year-old girl with epilepsy and autism from the village of Rzepiski in Podlasie region went missing on 5 October when she left home to go mushroom picking in a nearby forest. She was found after six days. Exhausted and hungry, she was taken to hospital. It turned out that her feet were frostbitten.’
Institutional	Concerns institutions, places or infrastructure related to autistic people, e.g. describes the activities of the foundation or the construction of a new facility to support the care of autistic people.	13.8% (186)	‘They put up the kindergarten quickly. After nine months, the kindergarten for autistic children in Gorzow Malyszyn became a reality.’ ‘No institution in the Starachowice district wants to run classes for adults with autism. Instead of thinking about rehabilitation they are forced to sit at home all day.‘’
Social functioning	The article describes the life and social interactions of autistic people, events in their local community, etc.	10.4% (140)	‘For this boy, his birthday is a sad day. His mother wants to change that. Gifts, wishes and the presence of loved ones - that’s how every person’s birthday should be. Some, however, can only dream about it. Adrian Nowicki, who suffers from Asperger’s syndrome, usually spends his birthday only in the company of his mother. Why? Because his peers have never used the invitation.’
Charity	Charitable activities for autistic people, such as fundraising.	6.7% (91)	‘Owners of muscle-powered vehicles will ride through the streets of Warsaw for the third time in the Social Bike Rally for People with Autism. They will be motivated not only by the desire to draw attention to the problems of people affected by this disease. The start of each participant will also bring tangible benefits to the sick community. For every kilometre they ride, the Orange Foundation will donate around PLN 3.5 to people with autism.’
Education of autistic people	Articles on the school system, curricula, school functioning or autistic students’ experiences of education.	6.6% (89)	‘Integration is not always best for the child. Deputy Mayor Wioleta Haręźlak believes that creating special classes for children with autism is a bad idea. Specialists are of a different opinion. The ‘Dalej Razem’ association from Zielona Góra plans to create a kindergarten and a school for children with autism. A similar facility has been operating for many years in, among others, Gorzów. The centre there has 54 persons under its care. Each child has their own therapist and individual teaching programme. Meanwhile, Wioleta Haręźlak, Vice-President of Zielona Góra, believes that children with autism must not be isolated. - Many of them do very well in ordinary schools, all they need is the right therapy,’ believes the vice-president.’
Family situation	The article describes the situation of an autistic person’s family.	6.5% (88)	‘Respite care not for autistic people and their families. By using a scale relating to physical disability, autistic people and their carers do not qualify for some of the benefits. The Ministry of Family, Labour and Social Policy is collecting applications for benefits for so-called respite care, i.e. emergency support for carers of people with disabilities. This is particularly the case for people who devote all their time to caring for a child or a loved one and have had to give up their careers. The aim of respite care is to enable them to attend to urgent matters, such as going to the authorities or even staying in hospital.‘
Other	Frame not mentioned in other codes.	6.2% (84)	
Culture and art	Art and cultural events created by autistic people or about autism and autistic people.	5.7% (77)	‘The Będzin theatre knows that the mission of the children’s stage is not only to stage fairy tales about dwarfs and princesses, but also to provoke conversation about everyday problems. (…) ‘Alien’ is not a story addressed to children affected by autism, but to their siblings and all children. It is an opportunity to talk about acceptance and tolerance towards otherness,‘ says Dariusz Wiktorowicz, director of the play and director of the theatre.’ ‘‘Theatre 21’ was established at Warsaw’s special school ‘Daj Szansę’ on Głogowa Street2B. It was founded by Justyna Sobczyk, a graduate of theatre pedagogy in Berlin and special education. The ensemble’s members are students and graduates of the school, people with Down’s syndrome and autism. Their repertoire includes original performances, developed during rehearsals. ‘Theatre 21’ is the only theatre of its kind in Poland.’
Economic and financial	It concerns, for example, funding for care or educational subsidies for autistic people, their families or schools.	3.4% (46)	‘There is still not enough of this help. Pensions, allowances, individual lessons, subsidies for rehabilitation… Carers of autistic patients can get help from several sources. In dramatic situations, they solicit money from community collections. Autism is a very ‘demanding’ disease. Karol’s mum, Mrs Elżbieta Duława, has had to give up her job to look after her son. Meanwhile, the expenses associated with Karol are enormous.’ ‘In a pile of papers they lose a child. Parents of autistic children are sounding the alarm. More and more often, officials who are insensitive to their plight are depriving them of their care benefits. Because? Since the children are not bedridden, the parents can go to work. Experts comment: - It is sad that we still talk so little about autism’
Celebrities	The article is about celebrities with autism, talking about it or who are connected to the autistic community.	2.5% (34)	‘I saw several films, starting with “Rain Man”, met with the charges of the Synapsis foundation. I worked with therapists and psychologists. I also realised then that these people needed support, both psychological and financial. At first, we used a mobile phone to record some videos in the metro, in a café. And then I played an autism sufferer on TV’ [interview with a Polish actor involved in a social campaign for people on the spectrum] ‘Agnieszka Chylińska [Polish rock/pop singer] hides it from everyone. The name of a certain illness was mentioned in her book. When Agnieszka Chylińska (44) said in interviews that ‘motherhood has done her in’, no one even suspected the truth behind it. (…) It was only on the occasion of the Women’s Strike that the juror of the programme ‘Got Talent‘ confessed that two of her children are “special needs” children.’
Misuse	Using the word ‘autism’ or ‘autistic’ in the wrong context, e.g. ‘architectural autism’ or ‘political autism’.	0.4% (5)	‘I have the impression that this building is detached from material reality to such an extent that the architecture turns into a stage set for the eye, aimed only at producing striking and memorable images,’ he admits. In his industry, the term ‘architectural autism’ is increasingly being used to describe such buildings. This is mainly due to the fact that in their design the greatest emphasis is placed on the visual experience. The others are overlooked, leading to the disappearance of the multi-sensory quality of the space. And architecture, at least in principle, should stimulate all the senses. Architectural autism is now - and worldwide - as popular a phenomenon as sky-high office buildings or giving a second life to post-factory halls.
**Overall tone**	**What is the overall tone of the article?**		
Negative	The article focuses on deficits, difficulties and problems.	41.6% (563)	‘Iris’s parents found out the truth on the internet. In an online test, their daughter ‘scored all red flags’. She didn’t respond to her name, avoided eye contact, was annoyed by small changes, didn’t imitate her peers, didn’t speak, and instead of playing with toys or enjoying the playground attractions, she counted the screws in cars and carousels… The medical diagnosis only confirmed Arabella’s suspicions - her daughter had profound autism. It took time for the parents to realise that Iris was ‘lost to them somewhere’. There was a long road ahead of them. They had to travel it to save their daughter. To find a thread of understanding with her. To break her out of her closed world. However, no doctor gave them a guarantee of success. Autism is still an unexplored disease. There are no sure prescriptions in it.’ ‘The capital of Warmia and Mazury region, like many cities around the world, has once again joined the campaign to raise awareness of the autism disease. It has been recognised by the UN as the most dangerous civilisation disease in the 21st century, next to AIDS and cancer.‘’
Neutral	The article describes facts without any explicit value judgements.	37.4% (506)	‘At Sienkiewicza Street autistics have their home. Therapists from the association Dalej Razem, which helps autistics and their families, will conduct classes in the historic villa on Sienkiewicza Street in Zielona Góra from July.’
Mixed	Contain both negative and positive elements in similar proportion.	14.3% (194)	‘In Denmark, people with autism, for example, work for a telecommunications company and assemble SIM cards. They are better at this than students, because they notice and catch errors that others miss,’ Sławomir Ulankiewicz, Polish representative of the Specialisterne Foundation, said yesterday. According to estimates, nearly 30,000 people (including 10,000 adults) are affected by autism in Poland. Depending on the severity of the disease, they suffer from motor hyperactivity, difficulties in communication. Instead, they have a unique ability to focus on details. Specialisterne has developed special techniques to recognise the vocational potential of people with autism.’
Positive	The article focuses on successes, or is challenging negative stereotypes and prejudices, describes autistic people in positive way, etc.	6.6% (89)	‘The only café of its kind in Poland. A beautiful initiative! As early as February, an extraordinary café will appear on the map of Warsaw. It is the only such place in Poland. Its employees will be people with autism. So renovations are starting in the premises, and the future employees are taking part in special training with therapists.’ ‘Many people complain that television today stupefies children instead of educating them. This claim is contradicted by the case of 13-year-old Brandon Williams. The boy gets his knowledge of life from cartoons. Unexpectedly, thanks to his fascination with the Sponge Bob cartoon, the autistic boy saved the life of his classmate!’ [Statement from a mother of a teenager on the spectrum] ‘I think Tomek is the kind of guy who has handled everything perfectly so far. I am full of admiration for him. This is not to my credit. He was born and raised with a great sense of self-confidence and such a high self-esteem that compensates for any shortcomings. Sometimes I have to put the brakes on him. (…) This year has been for him an accumulation of winning chances. He took the whole pot, he did it by himself, and he actually stunned us.
**Voices included**	**Who is speaking in the article? The main voice communicating relevant information on autism in the article.**		
Journalists	Journalists covering the story from a third-person perspective	44.7% (605)	
Multiple voices – not including autistic people	Several different parties speak out about autism in the article, but people on the autism spectrum are not among them.	19.7% (266)	
Parents	Parent/parents of a person on the spectrum speak in the article.	9.3% (126)	
Autism community activist	One or more activists representing autism-related community (e.g. NGO members) speak in the article.	7.7% (104)	
Health professionals	Broadly understood health professionals (doctors, psychologists, speech therapists etc.)	4.3% (58)	
Other voices	Voices not mentioned in other codes.	4.1% (56)	
Scientists	Scientists (university or other researchers conducting or commenting research on autism) speaking in the article.	3.9% (53)	
Multiple voices – including autistic people	Several different parties speak out about autism in the article, and people on the autism spectrum are among them.	3.5% (39)	
Celebrity	Celebrities, e.g. famous musicians speaking about autism.	1.4% (19)	
Lawyer	Lawyers / law experts speaking about autism.	0.6% (8)	
Other family members/friends	Family members (other than parents) and friends of a person on the spectrum speaking in the artice.	0.6% (8)	
Only autistic people	Autistic people as the only/main source of information.	0.2% (3)	
**Neurodiversity lens**	**Does the article mention neurodiversity**,** raise awareness and emphasize tolerance for difference**,** highlight potential of autistic people**,** etc.?**		
No		70.7% (956)	
Yes		29.3% (396)	‘We want to show that autism is not actually a disease. It is a condition. We want to show that autistic people, even though they are different and perceive the world differently, with the help and understanding of those around them can live, function and work normally. We want to show what autism is without stereotypes.’ ‘More and more movements for the rights of people with different disabilities are emerging. For example, autistic people who get on quite well in social interactions want autism to stop being considered a disease and to simply become an accepted difference. Autistic Pride Day, for example, is intended to serve this purpose.’ ‘Autistic people are employed just like other employees. They have contracts, assigned positions, responsibilities. Depending on their skills and the degree to which they function, they do different jobs. They have become specialists in their fields. They prepare buckwheat sticks, make individual components for therapeutic aids, print, laminate, cut, bind, prepare graphics and photos for the alternative communication system, create promotional videos. They have a job and everything that goes with it: responsibilities, problems, successes. Colleagues they like more or less, a boss and clients with their demands. Every day they overcome many of their weaknesses, they are constantly learning, but thanks to the work they do, they feel needed, appreciated.’
**Attention paid to ASC**	**How insightful the article analyses the topic of autism?**		
Briefly mentions ASC	E.g. an article just mentions about a person on the spectrum or mentions the word ‘autism’ listed among other conditions.	43.1% (583)	
Relevant but not leading thread	E.g. the story described is significantly related to autism or autistic people but does not analyse the topic in detail, does not go into what autism is or what life is like for autistic people.	36.7% (496)	
Fully dedicated to ASC	Discusses the issue of ASC in detail and/or is fully dedicated to the lives of autistic people.	20.2% (273)	
**Helpful information**	**Does the text contain helpful and reliable information about autism**, e.g.,** helps to better understand the reasons for autistic people’s behavior or their specific perception of the world?**		
No	No information / explanation is provided.	73.7% (996)	
Partially	One or a few situations described in the article are explained in a way that helps the reader understand its background, reasons or behavior, motivations etc.	14.1% (191)	‘In the course of our therapeutic work, I often have the opportunity to hear from parents of sick children that for their families New Year’s Eve fireworks are a nightmare. After such an experience, their kids simply don’t want to leave the house even for a week, so strong is the sense of danger caused by the sound of the explosions.’
Yes	All or most situations described in the article are explained in a way that helps the reader understand its background, reasons or behavior, motivations etc.	12.2% (165)	‘It is not surprising that such campaigns are carried out, since awareness, knowledge about autism and acceptance of autistic people in society are still not satisfactory. A survey of autistic people and their relatives showed that 77% of them find it stressful to leave the house and 52% avoid leaving the house at all. Not surprisingly: 86% of those surveyed did not identify a single place that would suit their needs. They are pushed to the margins of their lives more than anything by people’s reactions to specific behaviours: 60% were met with critical stares, 46% heard negative comments and 12% even behaved aggressively towards 12%. (…) A child with autism may find it difficult to interact and simply talk to others. Many are characterised by delayed speech development or do not speak at all. They often do not look at their eyes or respond to their name. They find it very difficult to understand the intentions and behaviour of people in their immediate environment and to imitate them. Autism can also manifest as a problem in expressing one’s own emotions and understanding inner experiences.’
**What should be changed?**	Does the article specify that it is autistic people that should be adapted to meet the requirements of the society or these are the social settings that should be changed to fit autistic people’s needs better (only if article mentions this issue*)?	*(*n* = 563)	
Autistic people	E.g. by therapeutical actions.	60.2% (339)	‘The parents began to fight for their son, going from doctor to doctor to find out what Borisek was ill with. It was only after a few weeks that the correct diagnosis was made - autism. Now every day the little boy is rehabilitated, but he does not speak. Brave mum does not give up and fights for her son with all her strength. The salvation for Borys is dolphin therapy. Dolphins can teach Boris to speak. The cheapest is implemented in the Crimea, but it still costs about 12,000 zloty.’
Environmental/social settings	E.g. by rising social awareness about the needs of autistic people or adjusting the intensity of the stimuli in the environment.	11.00% (62)	‘’Quiet hours’ at Carrefour have been introduced for people with disabilities. This particularly concerns customers with autism spectrum disorders and sensory hypersensitivity. The changes will make it easier for them to shop. With them in mind, the ‘quiet hours’ will also include slower checkout times. - ‘Through tests in two shops in Warsaw, we want to develop the most optimal model of this initiative for customers with sensory hypersensitivity.’‘
Both	Article mentions both options.	28.8% (162)	

Before the first coding, precise guidelines were developed regarding what a given category means and when an article should be assigned to it. Categories such as ‘neurodiversity lenses’ and ‘Briefly mentions ASC’ were defined. Like the coding categories, these guidelines were developed largely using previous research on press discourse on the autism spectrum, which significantly facilitated the coding process (see e.g. Jones & Harwood, Bie & Tang, Billawalla & Wolbring [11, 14, 18]. In the vast majority of cases, this method was sufficient to achieve agreement between coders. In the rare cases where their ratings differed significantly, the interpretation of a given category was established during the discussion. Assigning text to categories was mostly straightforward, e.g. the main frame of the article, the emphasis on the change of the person on the spectrum vs. the change of their environment, or the identification of who is speaking in the text was clear immediately after reading it.

In the case of categories requiring some interpretation, we used previously prepared guidelines, following the method of Jones & Harwood [14]). The use of the guidelines, along with the discussion of the few differences in the pre-coded articles, eliminated a few discrepancies between coders.

First, two coders assigned one hundred randomly chosen articles to predefined categories. The results were compared for inter-rater reliability, and a few discrepancies were discussed and resolved by making the definitions of selected codes more precise. Joint initial coding was performed to determine the extent of the principal investigator’s preconceptions and biases. As the differences between raters were marginal, exact compliance rates were not calculated. Minor discrepancies were resolved by making the definitions of the individual codes more precise. The PI coded the rest of the articles. If an article portrayed autism as a source of deficits and problems for autistic people, its tone was assessed as negative. If the text referred to successes and advantages or showed the possibilities of autistic people (e.g., the opening of a café run by autistic people or statements by parents highlighting the uniqueness of their children), the tone was described as positive. A large group of articles that contained both were coded as ‘positive and negative’. The tone was described as neutral when the text contained no explicitly or implicitly value-laden information.

#### Analysis

It is important to point out that the lead author of the manuscript is himself an autistic person, and therefore, the authors of this paper speak from the ‘engaged humanities’ perspective. Our research aims to recognize, highlight, and critique unfavorable power relations between the neurotypical majority and the autistic community (the community of autistic people, including self-advocates as distinguished from ‘autism community’ referring autistic people, their families and possible professionals engaged with supporting and advocating for autistic people and their families). Critical Discourse Analysis and Critical Autism Studies can show how the picture of autism is established within the media and who has the main say [38]. To clarify our position, it is also worth stating that the research was co-led by an autism community member, a parent of two autistic children, but not autistic himself. Our perspective is not anti-psychiatric, and we do not consider the ontological findings of psychiatry as harmful constructs. Nevertheless, it is critical of the deficit-laden and bio-reductionist psychiatric mainstream in the Polish context.

The social and medical discourses were contrasted for the sake of analysis only. We do not see them as contradictory but rather as dialectical. This simplification, however, may allow to present the main lenses through which autism is publicly perceived with better clarity. We also do not consider ‘scientific’ and ‘medical’ perspectives as identical and homogeneous. The current scientific literature contains multiple examples of positions that are not discriminating. Also, ‘medical’ does not necessarily mean ‘bad’ or ‘harmful’, yet the dominance of the medical perspective shapes public opinion about ASC people. Therefore, it should be identified, and its influence should be critically evaluated.

The comparison of the articles’ parameters between the group of autistic people (*n* = 42) and all other groups of speakers (*n* = 1,310) was not possible statistically due to their unequal size. These groups were, therefore, compared in terms of incidence only. When comparing articles presenting the first-person perspective of autistic people (*n* = 42) with health professionals (*n* = 58), we used a Chi2 test and Cramér’s V coefficient. We used SPSS (ver. 27.0) for statistical analysis.

## Results

### Main tone of the discourse (research questions 1–3)

Autism is primarily portrayed as a biomedical, negative, and deficit-laden phenomenon that should be addressed by bringing autistic people in line with the neurotypical majority, while their own views are considered irrelevant. The deficit-laden perspective, devoid of autistic people’s voices, was found in the discourse in general and among health professionals in particular.

**Fig. 1:**
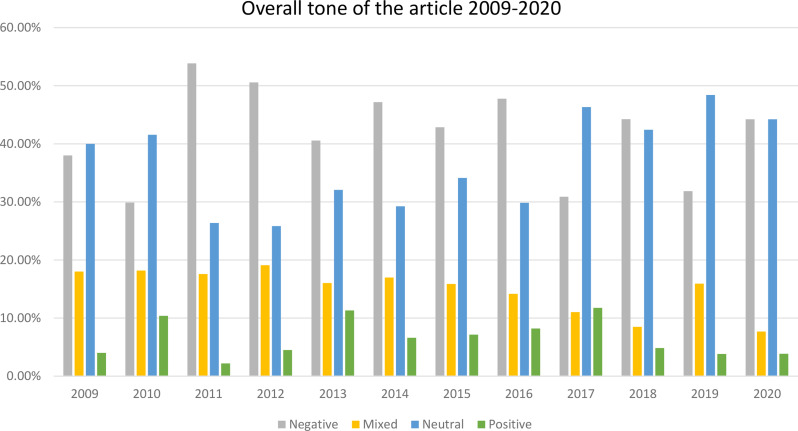
Overall tone of article – changes over 2009–2020

The overall tone of the discourse is mostly negative (41.6%) or neutral (37.4%) and only marginally positive (6.6%), with 14.3% of articles presenting a mixed view. Over the last decade, it changed a little toward the neutral tone ( see Fig. 1). The most popular frames are medical (22%), news and criminal cases (15.8%), institutional (places and infrastructure) (13.8%), with 10.4% of articles dedicated to the social functioning of autistic people and 6.7% to charity events (see Table 3).

**Fig. 2:**
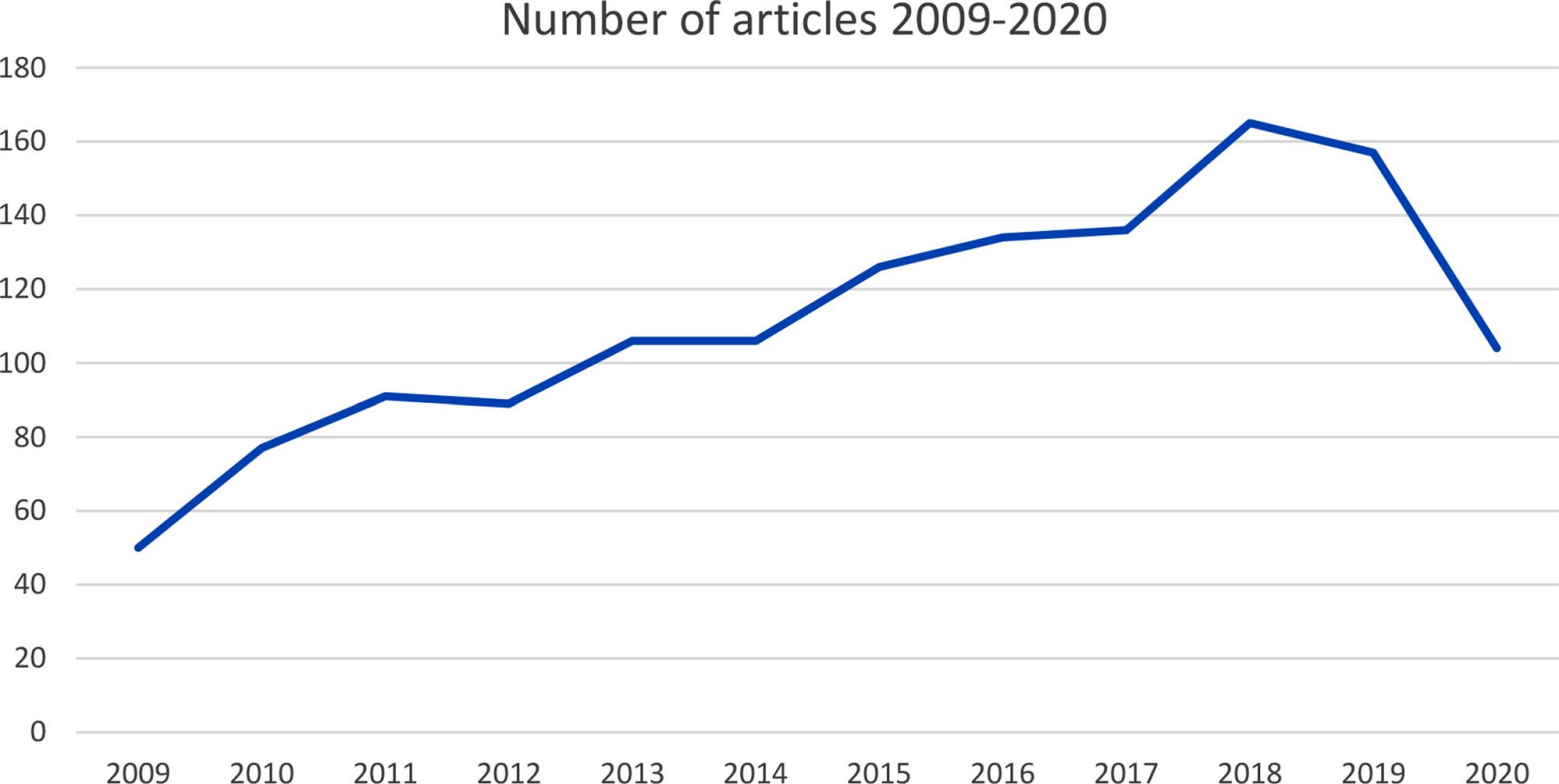
Increase in number of articles over time (2009–2020)

**Fig. 3:**
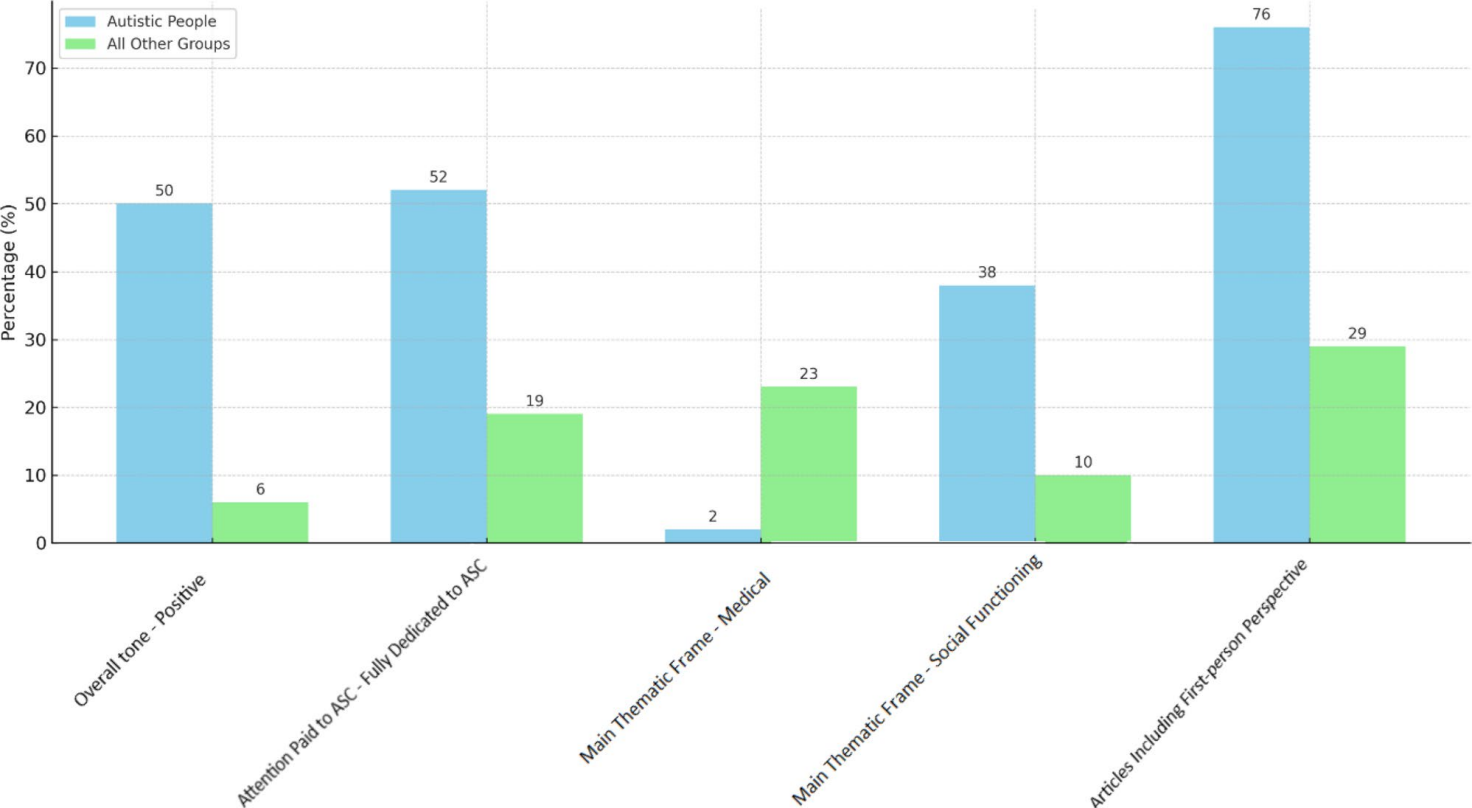
Comparison of selected categories between autistic people and all other groups

**Fig. 4:**
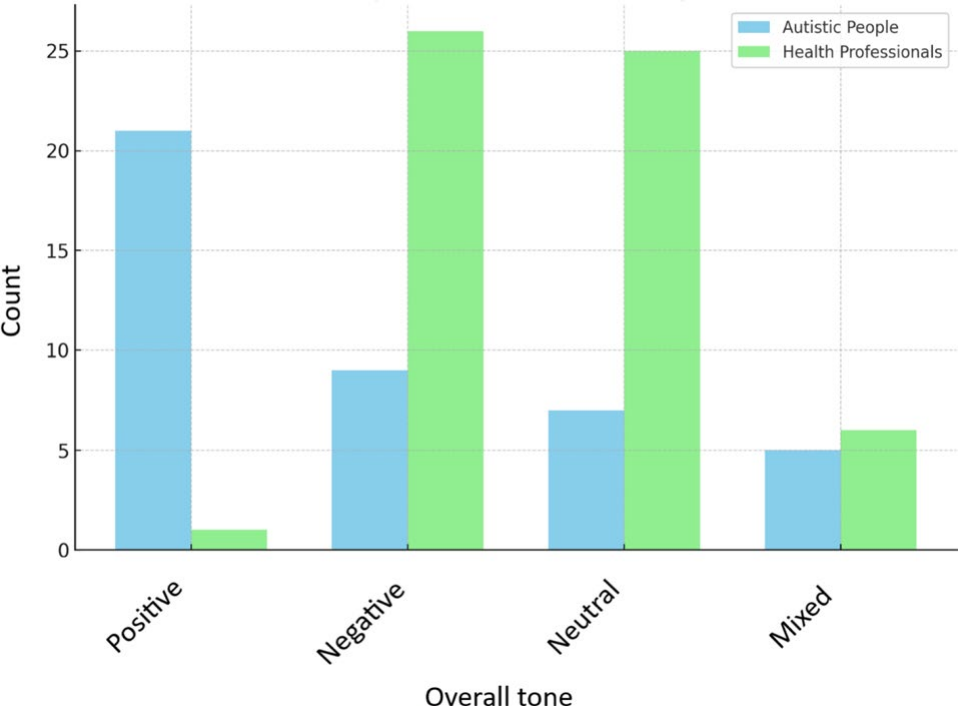
Discursive image of ASC – overall tone of the articles (autistic people vs. health professionals)

**Fig. 5:**
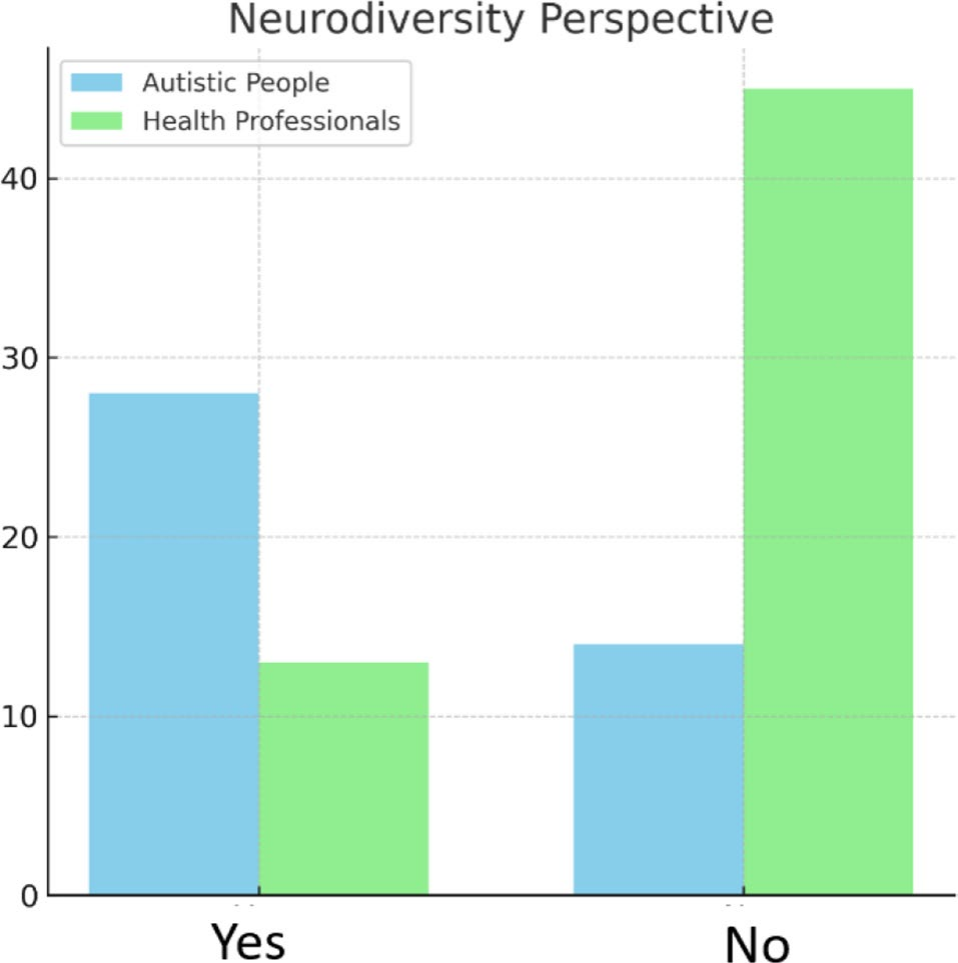
Discursive image of ASC – inclusion of neurodiversity perspective (autistic people vs. health professionals)

**Fig. 6:**
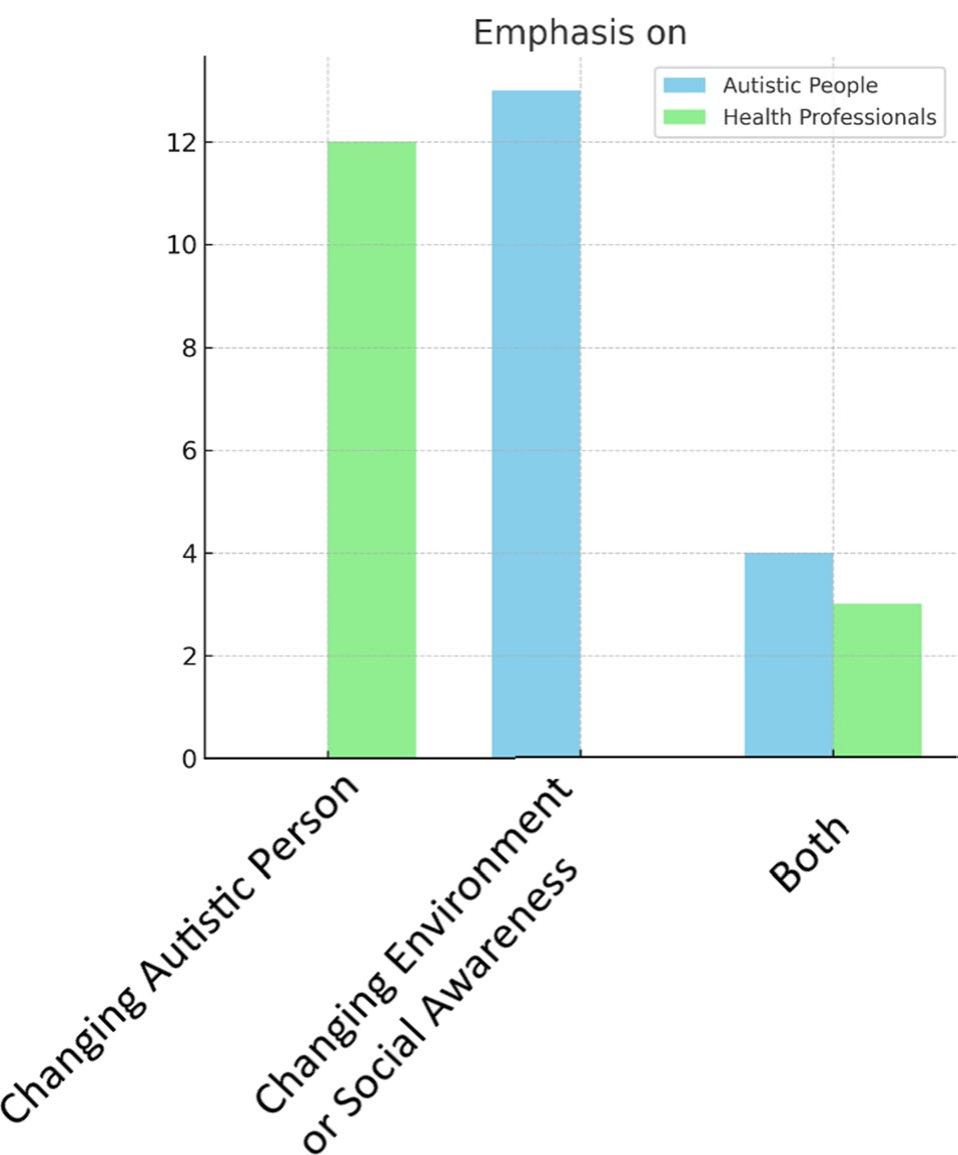
Discursive image of ASC – emphasis on changing: ASC person; environment or social awareness; both (autistic people vs. health professionals)

It is noteworthy that the number of articles devoted to autism in the analysed field increased significantly in the selected period, steadily increasing year on year between 2009 and 2018, before decreasing slightly in the following two years (2009 *n* = 50, 2018 *n* = 165, 2019 *n* = 157, 2020 *n* = 104, see Fig. 2).

This seems to be in line with the fact of increasing media interest in the topic of autism in various fields. This fact is all the more indicative of the need to take care of the quality of press discourse so that it does not cause stigmatisation and misinformation about autism and people on the spectrum.

**Table 4:** Discursive image of ASC - comparison of selected categories between autistic people and all other groups

		Autistic people (*n* = 42) No. (%)	All other groups, including health professionals (*n* = 1 310) No. (%)
Overall tone of the article	Positive	21 (50.00)	75 (5.72)
Attention is paid to ASC	Fully dedicated	22 (52.38)	243 (18.54)
Main frame	Medical	1 (2.38)	295 (22.51)
	Social functioning	16 (38.09)	125 (9.54)
First-person perspective of autistic people/their families	Yes	32 (76.19)	380 (29.00)

**Table 5:** Discursive image of ASC - comparison of selected categories between autistic people and health professionals

	Autism portrayed				Neurodiversity perspective		Emphasis on		
**Who speaks?**	Positively	Negatively	Neutrally	Both	Yes	No	Changing autistic person	Changing environment or social awareness	Both
Autistic people (*n* = 42)	21	9	7	5	28	14	0	13	4
Health professionals (*n* = 58)	1	26	25	6	13	45	12	0	3
	Chi2 (3) = 34,99, *p* < 0,001 V = 0,59				Chi2 (1) = 19,72, *p* < 0,001 V = 0,44		Chi2 (3) = 28,07, *p* < 0,001 V = 0,53		

Chi2 values refer to the comparison of the ‘Who speaks?’ category with the individual categories included in each column (‘Autism portrayed’, ‘Neurodiversity perspective’, ‘Emphasis on’)

#### Who speaks? (Research questions 4–7)

The public discussions on ASC are dominated by journalists (44.7% - as the only or main voice in the article) and multiple voices not including autistic people (19.7% - e.g., journalist, researcher, and social worker); next come parents (9.3%), autism community activists (7.7%), health professionals (4.3%), other voices (4.1%), scientists (3.9%), multiple voices including autistic people (3.5%), celebrities (1.4%), lawyers (0.6%), other family members and friends (0.6%). The least represented category were the articles including only the voices of autistic people (0.2%).

The voice of both autistic people and health professionals is marginal in terms of numbers, respectively 3.7% (when two categories: ‘multiple voices including autistic people’ and ‘only autistic people’ are combined) and 4.3%. Almost 74% of articles do not include any helpful and reliable information to help the reader understand the reasons of described behavior or nature of autism, and 85% of those directly speaking of ASC traits use negative terms such as antisociality, violence, irrationality, difficult behaviors, and fixation.

#### When ASC people speak, the tone of the discourse changes (research questions 8–10)

When autistic people speak, the overall tone of discourse becomes more positive, more articles are fully dedicated to the issue of ASC. Therefore autism is described in more detail, and more attention is paid to social functioning than to the medical perspective (see Fig. 3). Moreover, the first-person and family perspective appears more often (e.g., autistic people talking not only about autism but about their general experience of the world, appearing in the role of epistemic authorities), thus autism is seen as a more complex and heterogeneous phenomenon (see Table 4).

Does a similar modification of the discursive message come along with the voice of health professionals? Compared with autistic people, health professionals portray autism more negatively (category: overall tone of the article for both groups including autistic voices vs. health professionals, *p* < 0,001, V = 0,59) (see Fig. 4), and less often describe it from a neurodiversity-related perspective (category: neurodiversity lens for both groups including autistic voices vs. health professionals, *p* < 0,001, V = 0,44) (see Fig. 5). The neurodiversity-related perspective is defined here by the emphasis on tolerance of functional differences. Also, health professionals more often emphasize the need to change autistic individuals to fit into the neurotypical society rather than changing the environment or social awareness (category: what should be changed? for both groups including autistic voices vs. health professionals, *p* < 0,001, V = 0,53, Table 5) (see Fig. 6).

Although the number of articles giving voice to autistic people has been increasing over the analyzed period (2009: *n* = 0; 2020: *n* = 7), it is still significantly low. It also does not impact the overall tone of the discourse on ASC.

It is worth noting, however, that while the discourse as a whole may still be harmful, some changes for the better are taking place in the long run. The medical narrative loses some popularity, and interest in the sphere of social functioning and family situation of autistic people grows. The number of articles in the ‘news and crimes’ category also increases, which may imply a perpetuation of the stereotype of autistic people as dangerous or dependent (for example, numerous articles have dealt with missing autistic people).

## Discussion

### Summary

To date, the image of autism in Polish newspapers has not been studied, so our study fills an important gap by showing and analysing the discursive image of autism in a new cultural context. Two perspectives dominate Polish media discourse on autism. Firstly, the ‘bio-medical’ perspective, related to the widespread medicalization of autism and its positioning as an undesirable medical condition that needs to be ‘fixed’. Second, a ‘socio-negative’ perspective that portrays autistic people as ‘victims’ of autism, mainly through the prism of the difficulties they face in social functioning. Notably, the suggested solution to these difficulties emphasizes changing autistic people rather than the environment and social awareness. The achievements of autistic people are often portrayed as ‘unusual’ and ‘amazing’, even if these achievements would not be considered a success for most neurotypical people. Nevertheless, the voice of the autistic community and self-advocates is becoming more powerful and it is increasingly heard. The mainstream media give voice to self-advocates and their families, who create more of insider’s content.

### Comparison with other countries

The results align with findings from previous studies conducted in other countries such as the USA, UK, Australia, and China. One significant similarity is the marginalization of autistic voices. Like in our study, where only 3.7% of the articles featured first-person perspectives from autistic individuals, previous research (e.g., Bie & Tang [18, 39]; Huws & Jones [14], Baroutsis et al. [21] Mittmann et al. [22] has shown that autistic voices are similarly underrepresented in media narratives across different contexts. This absence reinforces stereotypes and contributes to a one-sided portrayal dominated by non-autistic voices, including health professionals, journalists, and family members.

Another point of convergence is the predominance of negative and deficit-laden portrayals of autism. Our study found that 41.6% of the articles had a negative tone, focusing on deficits and challenges faced by autistic individuals, while similar findings were noted in research by Wolbring & Mosig [11] and Jones & Harwood [14], or Karaminis et al. [19] where autism was frequently framed through a medicalized lens as a disorder to be treated or corrected. Both our study and prior research emphasize how this type of framing marginalizes the neurodiversity perspective and perpetuates harmful stereotypes, positioning autistic people as needing to conform to neurotypical standards rather than celebrating their unique contributions.

Karaminis et al. in a study performed on a corpus of nearly 24,000 UK press texts showed that the dominant narrative depicted autism through the lens of adversity (medical frame, negative language, emphasis on disability) and was biased towards showing boys. However, researchers noted a gradual shift in the narrative towards difference-laden rather than deficit-laden and a more balanced representation in terms of age and gender of people on the autism spectrum. This is in line with the findings of Lewin & Akhtar [24] who showed that despite the apparent positive change in the autism discourse, some negative elements have remained unchanged for years.

Lastly, our study and previous analyses show that when autistic individuals’ voices are included, the tone of the discourse becomes more positive and nuanced. Articles that feature first-person testimonies often highlight the social aspects of autism and advocate for greater tolerance and understanding, shifting away from the predominantly medicalized view. This suggests that increasing autistic representation in media can play a critical role in reshaping the press representation of autism.

However, our research is the first to show how the stereotypical perspective, including that of health professionals represented in the Polish press titles, changes when the discourse on ASC includes first-person testimonies of autistic people. Since more open and less deficit-driven and medicalized positions on autism are less represented in the former Eastern bloc countries, the focus on the regional perspective is a vital asset of our study.

### The role of the media in perpetuating negative stereotypes of autism

Media reports play a crucial role in shaping and influencing public discourse by framing the way issues are discussed and perceived by society, e.g. through agenda-setting [28] or framing [29]. The media determine which topics are given prominence, and how they are interpreted by the public opinion. For instance, when autism is consistently framed through a deficit-based or medicalized lens, this shapes societal attitudes and reinforces stereotypes, often perpetuating negative perceptions. Moreover, by selecting particular voices—such as experts over individuals from marginalized groups like autistic people—the media determines who is seen as authoritative on these topics. This influence extends beyond individual perception and can impact public policy and social norms [31]. While this voice might be powerful enough to change public awareness (both among neurotypical audiences and among autistic people themselves), it is still, unfortunately, marginal. There is great potential to change this situation with the increasing presence of self-advocating young people (such as the aforementioned Aware Youth Club), who break through various ‘professional discourses’ on autism (including those created by self-advocates). By showing their own example, self-advocates do not replicate the pattern of ‘victim’ and ‘object of medicalization’.

Why is the overall tone of the discourse mostly negative? Why is autism so often portrayed as a negative and deficit-laden phenomenon? One reason might be that journalists and media corporations are often attracted by topics that are sensationalist or describe real and potential threats - both to the general public and to the minority groups described. While this attitude may stem from a sense of duty to report on risks [40, 41], it can translate into biasing the tone of discourse on autism, or mental health more broadly, towards the dramatic [42]. Journalists often prioritize sensational stories to attract larger audiences, focusing on emotional appeal. This is driven by commercial pressures and competition for attention in a crowded media environment [43, 44]. Many articles have titles designed to attract attention at all costs (e.g. “THE DRAMA of Monika Richardson”, a Polish actress and journalist with an autistic son). Even though the protagonists portrayed autism in a neutral or even positive light, the comments or titles added by journalists were often intended to arouse strong emotions. All the analyzed titles were popular daily newspapers, and it is unlikely that the same discursive tone would re-appear in a more professional fraction of press titles.

It should be noted, however, that journalists are not the only culprits here. Readers themselves often prefer - contrary to their statements - negative to positive news [45]. There is also evidence showing that humans tend to react more strongly to negative news content, which may account for the prevalence of negative news coverage [46].

### The role of health professionals

The fact that health professionals in the Polish public discourse portray autism more negatively than autistic people is quite unfortunate. It may be that they are not representative of the population of experts. This research also indicates that health professionals who speak publicly about autism neither emphasize tolerance of autistic people’s functional differences nor highlight their potential. They rather underline the need to change autistic people to fit them into the neurotypical society. It might also be because the experts belong to the ASC discourse and both create it and receive its message as readers.

Experts, including mental health experts, partake in different discourses and are not immune to stereotyping that affects their decision-making [47–50]. Thus, considering the backward situation of Central Eastern Europe in terms of the propagation and distribution of alternative models of understanding autism and neurodiversity, this might affect their decision-making. For example, a survey of 183 autistic people conducted by the JiM Foundation [51] showed that 65% of them declare that their therapists are often guided by stereotypes and even undermine their diagnosis. In addition, many autistic people feel neglected and ignored. Thus, the discursive exclusion of autistic people as sources of knowledge about themselves re-appears in the clinical evaluation. Also, the discursive dominance of the biomedical frame and the under-representation of social perspective corresponds to an excessive focus on the physical health of autistic people visible in experts’ opinions (publicly accessible through the Polish Judgments Portal of the Common Courts).

### The public role of autistic individuals

It has become commonplace to say that we should carefully listen to our patients. Our research shows, on a large dataset, how the discursive meanings might shift when autistic people are listed to. Their testimonies change the public image of autism to more nuanced and realistic, and may also affect background knowledge and tacit understanding of ASC. Even if these findings need to be tested further, they provide important guidance on reshaping the public discourse on ASC.

We are aware that autistic people who are allowed to speak in public are not representative of the full spectrum of positions within autistic or health professionals community (this issue is vividly discussed in the literature [52, 53]. Yet, even if the autistic voices in question are not representative, they still shape the public discourse. It is important to remember that the broader ‘autism community’, whose voice should be widely considered in all discourses on autism, is an extremely diverse group. It includes people on the spectrum, their families, and various foundations supporting autistic people, fighting for their rights or lobbying for the introduction of less discriminatory legislation, including at the governmental level. Each of these groups has a slightly different perspective on the issue, different methods and goals, and each consists of factions with different needs (such as autistic adults and children, people who function on a day-to-day basis independently, and those who require more support, etc.). It is thus important to remember that we see only a part of the landscape.

We conclude that experts should carefully reflect on the quality of the information in the prevalent discourses, as it may become part of their background knowledge. The inclusion of autistic people’s voices is critical to changing public opinion and, indirectly, clinical judgment.

### Methodological implications of the neurodiverse interpretative framework

The results suggest that restoring a voice to people on the autism spectrum positively changes the discourse. First-person experience is not a mere ‘marginalized voice’ but an important emancipatory and discourse-shaping factor as well as an interpretative factor in this research. Adopting that neurodiverse perspective was possible by composing the research team of people with different neurotypes, drawing on their own experience and that of the autistic community [54, 55]. The combination of autistic and neurotypical perspectives allowed us to approach the discourse analysis with heightened sensitivity to different narrative frames and social representations of autism. The autistic perspective, in particular, was crucial in identifying subtle, often implicit, forms of bias and deficit-framing within the media narratives that a neurotypical-only analysis might overlook [56]. This complementary approach enriched the analysis process, ensuring a more nuanced evaluation of the articles, leading to a fuller understanding of how autism is portrayed in the Polish press. By emphasizing both deficit-based and strength-based perspectives, we were able to highlight not only the exclusion of autistic voices but also the potential for media to reinforce or challenge stereotypes. Autistic perspective added sensitivity regarding issues that the neurotypical researchers might have missed, e.g. subtle biases or misinterpretations that might have been considered non-stigmatizing.

‘Neurotypical’ analysis might interpret the fact that only 3.7% of the analyzed media articles included the first-person perspective of autistic people as a reflection of the overall lack of representation, which is an observable trend in many social discourses. However, the autistic perspective adds a crucial layer of understanding here: the very act of marginalizing autistic voices in favor of non-autistic speakers reinforces a harmful narrative where autistic people are treated as objects rather than active agents in their own lives. This implicit silencing perpetuates the idea that autistic individuals lack credibility or the ability to speak about their own experiences.

Furthermore, many of the articles coded as ‘neutral’ focused on autism from a purely medical perspective, describing it primarily as a condition that requires intervention to bring autistic individuals in line with neurotypical standards. While a neurotypical researcher might view this as standard medical discourse, the autistic perspective reveals that this framing inherently pathologizes autism by emphasizing deficits rather than strengths or alternative ways of experiencing the world. For example, articles describing therapies and interventions without addressing the social or environmental changes needed to accommodate autistic people can seem non-stigmatizing to neurotypical readers. Yet, they subtly reinforce the idea that autistic individuals need to be “fixed” to fit into society.

In some articles, autistic individuals are portrayed positively, but only when achieving something that is considered remarkable or “extraordinary” by neurotypical standards, such as excelling in a particular talent or overcoming challenges. These articles could be interpreted as purely positive, celebrating the success of autistic individuals. However, the autistic perspective highlights that such portrayals can be subtly stigmatizing because they imply that autistic people are valuable or worthy of recognition only when they perform beyond societal expectations. This framing reinforces the stereotype that autism is a condition that must be “overcome” and positions the achievements of autistic individuals as rare exceptions rather than validating and accepting their everyday experiences and diverse abilities as inherently valuable. This perspective is also crucial to understanding how even seemingly positive representations can contribute to stigmatization by setting unrealistic standards for autistic individuals.

### Limitations

Several limitations to this study should be acknowledged. First, the ability to generalize the results is constrained by the specific focus on Polish media. While the findings provide valuable insights into portraying autism within a particular cultural and geographical context, they may not directly apply to other countries with different media landscapes, cultural values, or approaches to autism. Additionally, the research exclusively examines press discourse, which overlooks other influential media forms, such as television, radio, and social media. Moreover, the autism community and discourse landscape have been undergoing significant changes in recent years, driven by the growing influence of the neurodiversity movement and activism. This study, covering media from 2009 to 2020, may not fully reflect the impact of these developments. The dynamic nature of autistic advocacy suggests that further research is needed to track how these evolving movements influence media portrayals and public perceptions over time.

## Conclusions

- Negative Portrayal of Autism: The study found that autism is predominantly portrayed as a negative, deficit-laden phenomenon within the Polish press. There’s a focus on aligning autistic individuals with neurotypical standards, often sidelining their perspectives.
- Tone Distribution: The overall tone of articles was mostly negative (41.6%) or neutral (37.4%), with a smaller proportion being positive (6.6%) or having a mixed tone (14.3%).
- Dominant Themes: The most common frames for discussing autism were medical issues (22%), followed by news and criminal cases (15.8%), and institutional, place, and infrastructure-related issues (13.8%).
- Voices Represented: Journalists were the predominant voice (44.7%), with minimal representation from autistic individuals themselves (3.7%) and health professionals (4.3%).
- Impact of Autistic People’s Voices: When articles included the perspectives of autistic individuals, the tone was more positive, and autism was discussed with more nuance, focusing on social functioning rather than medical aspects.
- Health Professionals’ Perspectives: Health professionals tend to portray autism more negatively compared to autistic individuals and emphasize the need for autistic people to conform to neurotypical society rather than advocating for societal or environmental adjustments.
- Increasing Visibility of Autistic Voices: Although still limited, the number of articles featuring first-person accounts from autistic individuals has been growing, indicating a slow but positive change in discourse.
- Biomedical vs. Socio-Negative Perspectives: The discourse is largely split between viewing autism as a medical issue to be fixed and seeing autistic people as victims of their condition, needing to adapt to societal norms.

## Data Availability

The datasets generated during and/or analysed during the current study are available from the corresponding author on reasonable request.

## Footnotes

1 More frequent attempts to describe autism as a condition of the body (ASC, autism spectrum condition) rather than a disorder can be observed. This is a way of perceiving autism that moves away from a rhetoric pathologising it towards a rhetoric that emphasises the variety of conditions of our organisms (Wright, Spikins & Pearson 2020; Baron-Cohen 2015).

2 Research conducted in the UK (Kenny 2016) on a large sample of 3470 respondents showed that individuals on the autism spectrum themselves prefer the term “autistic” – which is why we adopted this terminology.

## Abbreviations

ASC: Autism Spectrum Condition
